# Influence of blinding on treatment effect size estimate in randomized controlled trials of oral health interventions

**DOI:** 10.1186/s12874-018-0491-0

**Published:** 2018-05-18

**Authors:** Humam Saltaji, Susan Armijo-Olivo, Greta G. Cummings, Maryam Amin, Bruno R. da Costa, Carlos Flores-Mir

**Affiliations:** 1 Orthodontic Graduate Program, School of Dentistry, Edmonton Clinic Health Academy, University of Alberta, 11405-87 Ave, Edmonton, AB T6G 1C9 Canada; 2 Faculty of Rehabilitation Medicine, University of Alberta, Edmonton, AB Canada; 3 Faculty of Nursing, University of Alberta, Edmonton, AB Canada; 4 Division of Pediatric Dentistry, School of Dentistry, University of Alberta, Edmonton, AB Canada; 5 Department of Physical Therapy, Institute of Primary Health Care (BIHAM), Florida International University, Miami, USA; 6 University of Bern, Bern, Switzerland; 7 Division of Orthodontics, School of Dentistry, University of Alberta, Edmonton, Canada

**Keywords:** Randomized controlled trial, Meta-analysis, Research methodology, Study quality, Bias

## Abstract

**Background:**

Recent methodologic evidence suggests that lack of blinding in randomized trials can result in under- or overestimation of the treatment effect size. The objective of this study is to quantify the extent of bias associated with blinding in randomized controlled trials of oral health interventions.

**Methods:**

We selected all oral health meta-analyses that included a minimum of five randomized controlled trials. We extracted data, in duplicate, related to nine blinding-related criteria, namely: patient blinding, assessor blinding, care-provider blinding, investigator blinding, statistician blinding, blinding of both patients and assessors, study described as “double blind”, blinding of patients, assessors, and care providers concurrently, and the appropriateness of blinding. We quantified the impact of bias associated with blinding on the magnitude of effect size using a two-level meta-meta-analytic approach with a random effects model to allow for intra- and inter-meta-analysis heterogeneity.

**Results:**

We identified 540 randomized controlled trials, included in 64 meta-analyses, analyzing data from 137,957 patients. We identified significantly larger treatment effect size estimates in trials that had inadequate patient blinding (difference in treatment effect size = 0.12; 95% CI: 0.00 to 0.23), lack of blinding of both patients and assessors (difference = 0.19; 95% CI: 0.06 to 0.32), and lack of blinding of patients, assessors, and care-providers concurrently (difference = 0.14; 95% CI: 0.03 to 0.25). In contrast, assessor blinding (difference = 0.06; 95% CI: -0.06 to 0.18), caregiver blinding (difference = 0.02; 95% CI: -0.04 to 0.09), principal-investigator blinding (difference = − 0.02; 95% CI: -0.10 to 0.06), describing a trial as “double-blind” (difference = 0.09; 95% CI: -0.05 to 0.22), and lack of an appropriate method of blinding (difference = 0.06; 95% CI: -0.06 to 0.18) were not associated with over- or underestimated treatment effect size.

**Conclusions:**

We found significant differences in treatment effect size estimates between oral health trials based on lack of patient and assessor blinding. Treatment effect size estimates were 0.19 and 0.14 larger in trials with lack of blinding of both patients and assessors and blinding of patients, assessors, and care-providers concurrently. No significant differences were identified in other blinding criteria. Investigators of oral health systematic reviews should perform sensitivity analyses based on the adequacy of blinding in included trials.

**Electronic supplementary material:**

The online version of this article (10.1186/s12874-018-0491-0) contains supplementary material, which is available to authorized users.

## Background

As evidence-based practice has grown over the past two decades, there has been a consistent generation of new randomized controlled trials (RCTs) and systematic reviews in medicine and dentistry. Currently, thousands of RCTs and meta-analyses of these trials are published every year to guide healthcare professionals in their evidence-based decisions in clinical practice. In the field of dentistry alone, nearly 50 new clinical trials and 20 systematic reviews are published every month [1–3]. These trials and systematic reviews, in turn, support much of the treatment modalities and treatment recommendations in dental practice based on the current best-identified evidence. While RCTs, the building blocks of systematic reviews and meta-analyses, are considered to provide reliable evidence for dental decision making, RCTs are susceptible to bias (underestimation or overestimation of treatment effect size (ES) estimates) due to limitations in their design, conduct, and reporting [4, 5]. For results and outcomes of RCTs to be generalizable and valid to specific patient subsets, they need to be properly designed, carefully conducted, and accurately reported to a standard that warrants the implementation of their results [4, 6].

Blinding (or “masking”) has been recognized as an important criterion of high methodological quality, particularly with respect to internal validity of RCTs [7]. Blinding is broadly used in a trial to prevent performance bias (blinding of participants and care providers) and detection bias (blinding of assessors) [8–10]. Blinding can be applied at numerous levels of a trial, including participants, outcome assessors, care providers, data analysts, or other personnel. Thus, several terms (e.g., single-, double-, or triple-blind) have been used to describe blinding types [6, 11, 12]. However, the use of these terms has been inconsistent among research groups, and this contributed to conceptual and operational ambiguity. While appropriate blinding can reduce performance and detection biases, it is not always feasible to apply blinding in a trial, particularly in an RCT that involves surgical or device interventions such as oral surgery and orthodontics, as participants are often aware of the type of intervention they are receiving. The appropriateness of blinding depends on factors such as type of outcome examined (e.g., objective vs. subjective) [10] and type of intervention applied (e.g., surgical vs. drug) among others. For example, it is more difficult to implement blinding in RCTs of surgical interventions than to implement blinding in RCTs of drug interventions in which trial investigators can use placebo medications to attain adequate blinding [13].

Published meta-epidemiological studies focused on the blinding domain have found potential associations between treatment ESs and blinding of participants [14–19], care providers [15–17, 19], assessors [15, 17–21], and “double blinding” [22, 23]. While those meta-epidemiological investigations were conducted within numerous health fields, the value of their conclusions may be limited, based on numerous factors, when generalized to other healthcare fields. These factors include a failure to evaluate continuous outcomes because of a preference for assessing dichotomous outcomes [15, 21, 23], emergence of inconsistent methodological findings associated with treatment ESs [15, 17, 22], and the study being “underpowered” [24] by lacking adequate sample size, which is needed to properly quantify bias in RCTs. More notably, meta-epidemiological studies have reported that the extent of bias in the treatment ES associated with blinding varied across different medical fields as well as across different types of intervention [17, 24].

To date, no meta-epidemiological study has examined bias related to blinding in RCTs within any oral health subspecialties or scope of practice in dentistry. Therefore, it is unclear whether the previously mentioned conclusions hold true in the field of oral health research where blinding is sometimes difficult or not feasible, especially in oral health RCTs involving surgical or device interventions, such as orthodontic trials.

Thus, our specific research questions were: (1) Do oral health RCTs with adequate blinding of participants, outcome assessors, and health care providers yield different treatment ESs than trials with lack or unclear blinding? (2) Do specific nonmethodological meta-analysis characteristics (e.g., dental specialty, type of treatment, type of outcome [objective vs. subjective], magnitude of the treatment ES estimate, heterogeneity of meta-analysis) modify the association between blinding and treatment ES estimates? Findings generated from this work could be used to improve the conduct and reporting of oral health RCTs.

## Methods and analysis

This study is part of a large meta-epidemiological study that investigates the association between methodological characteristics and treatment ES estimates in oral health RCTs. The protocol for this meta-epidemiological study was registered on PROSPERO (CRD42014014070), and published a priori [25].

### Literature search

We conducted a comprehensive literature search using six electronic databases (PubMed, MEDLINE, EMBASE, ISI Web of Science, Evidence-Based Medicine Reviews–Cochrane Database of Systematic Reviews, and Health STAR) from database inception to May 2014. A health sciences information specialist assisted in planning the search strategy which included a combination of index terms and keywords related to systematic reviews and oral health. The search strategy for each database can be found in Additional file 1: Appendix 2. We also searched the American Dental Association (ADA)–Evidence-based Dentistry database [26] and hand-searched the reference lists of potentially relevant studies identified in the main search, which focused on quality of systematic reviews in oral health. We did not restrict searches to English language nor did we limit them by other means.

### Inclusion and exclusion criteria

Two independent reviewers (H.S., M.A.) with dental research and clinical backgrounds screened titles and abstracts retrieved. Abstracts deemed to meet inclusion criteria were selected, and then full text reports of these and those that lacked sufficient information in the abstract were retrieved for screening. The same assessors independently determined final eligibility of full texts; discrepancies were resolved through consensus.

We included meta-analyses if they met the following predefined eligibility criteria: the meta-analysis (1) was in the field of oral health research and examined a therapeutic intervention related to treatment, prevention, or rehabilitation of dental, oral, or craniofacial diseases [27, 28]; and (2) examined at least one continuous outcome and included a minimum of five randomized trials with quantitative data of treatment ES estimates.

We subsequently selected RCTs included in the selected meta-analyses that met the following predefined eligibility criteria: (1) the design was reported to be an RCT where findings were reported in a way that allowed for calculation of treatment ES estimates; (2) the comparison was between an intervention versus a placebo, with no treatment control, or standard care (trials with a comparison of one active intervention versus another active intervention and there was no clear direction of which intervention was superior were excluded); and (3) the trials examined a therapeutic intervention related to a dental specialty recognized by the American Dental Association (ADA) [28].

### Data extraction

A panel of five reviewers from diverse health research areas (H.S., C.H., J.S., J.F., S.A-O.) carried out data extraction. To ensure consistency during data extraction, two team members (H.S.; S.A-O) conducted reviewer training; the review panel evaluated 10 randomized trials not included in the final set of trials and then discussed them to achieve consistency. A similar reviewer training process was conducted in other studies performed by the same research team [29, 30]. Data extraction was performed in duplicate, that is, two assessors independently carried out data extraction, with consensus meetings employed to resolve any disagreement. One assessor with an oral health research background (H.S.) performed complete data extraction (*n* = 540, 100%) while another assessor (either C.H., J.S., or J.F.) with a health sciences (non-oral health) research background acted as a second assessor. The two assessors conferred with a third assessor (S.A-O.) if agreement could not be reached, to achieve complete consensus. Only consensus data were used for statistical analyses. A structured and pilot-tested data extraction template designed in a Microsoft Office Access database was used for data extraction.

The primary outcome reported for each review was used as the primary outcome for our analysis. Alternatively, the primary outcome for the analysis was determined as the outcome associated with the meta-analysis that involved the largest number of trials (in case the review’s primary outcome was binary, not clearly stated, or the quantitative analysis associated with the outcome included less than five trials). Details from each included randomized trial and meta-analysis were extracted; the following elements were extracted: means, standard deviations, sample sizes, publication year, dental specialty (e.g., dental public health, endodontics, periodontics, oral medicine and oral pathology, oral and maxillofacial surgery, prosthodontics and restorative dentistry, orthodontics and dentofacial orthopaedics, and pediatric dentistry), primary outcome assessed, type of comparison in a review, number of included trials in a review, trial design (e.g., parallel, split-mouth, crossover, and factorial), type of outcome in a trial (e.g., drug vs. nondrug or subjective vs. objective [23]), and number of centers in a trial (e.g., multicenter vs. single center). To classify the type of comparison, we used the classification of the comparison implemented in the quantitative analysis reported in the review (e.g., treatment vs. control).

To assess risk of bias associated with blinding in the selected randomized trials, we applied nine blinding-based criteria (see Table 1), namely: patient blinding (blinding of participants allocated to interventions), assessor blinding (blinding of data collectors), care-provider blinding (blinding of dental clinicians and/or therapists who provided the interventions), investigator blinding (blinding of the principal investigator), statistician blinding (blinding of the data analyst), blinding of both patients and assessors, study described as “double blind” (by trial investigators), blinding of patients, assessors, and care providers concurrently, and the appropriateness of blinding (blinding that was properly implemented within the trial’s components according to the primary outcome).

**Table 1:** Guidelines for quality assessment of included trials [7, 31–37, 63]

Items /Definitions	Yes	No	Unclear
Performance Bias			
Patient blinding [39]: Was knowledge of the allocated intervention adequately prevented during the study? “Blinding of patients is a must when outcomes are subjective or self-reported. When Outcomes are measured by an assessor, then assessors should be blinded to group allocation. When Outcomes are automated (there is no assessor involved) then, blinding of participants or assessors is not an issue.”	Any one of the following: No blinding or incomplete blinding, but the review authors judge that the outcome is not likely to be influenced by lack of blinding (Automated outcome or administrative); Blinding of participants and key study personnel ensured, and unlikely that the blinding could have been broken; Objectives automatized outcomes coming from databases or hospital register office.	Any one of the following: No blinding or incomplete blinding, and the outcome is likely to be influenced by lack of blinding; Blinding of key study participants and personnel attempted, but likely that the blinding could have been broken, and the outcome is likely to be influenced by lack of blinding.	Any one of the following: Insufficient information to permit judgement of ‘Low risk’ or ‘High risk’; The study did not address the issue of blinding.
Blinded therapist/care-provider	The study describes in the title, abstract, or text that the therapists/care-providers were blinded. The blinding was appropriate.	The study describes in the title, abstract, or text that the therapists/care-providers were not blinded, or because of the nature of the intervention (e.g., exercise prescription or supervision, etc.), the therapist could not be blinded.	There is insufficient information to permit a judgment.
Blinded principal-investigator	The study describes in the title, abstract, or text that the investigator was blinded. The blinding was appropriate.	The study describes in the title, abstract, or text that the investigator was not blinded.	There is insufficient information to permit a judgment.
Blinded statistician	The study describes in the title, abstract, or text that the statistician was blinded. The blinding was appropriate.	The study describes in the title, abstract, or text that the statistician was not blinded.	There is insufficient information to permit a judgment.
Detection Bias			
Assessor blinding [39]: Was knowledge of the allocated intervention adequately prevented during the study? Detection bias due to knowledge of the allocated interventions by outcome assessors.	Any one of the following: No blinding of outcome assessment, but the review authors judge that the outcome measurement is not likely to be influenced by lack of blinding; Blinding of outcome assessment ensured, and unlikely that the blinding could have been broken.	Any one of the following: No blinding of outcome assessment, and the outcome measurement is likely to be influenced by lack of blinding; Blinding of outcome assessment, but likely that the blinding could have been broken and the outcome measurement is likely to be influenced by lack of blinding.	Any one of the following: Insufficient information to permit judgement of ‘Low risk’ or ‘High risk’; The study did not address the issue of blinding.
Detection/Performance Bias			
Blinding of both patients and assessors) [39]	Both patient blinding and assessor blinding were judged as having low risk of bias.	Both patient blinding and assessor blinding were judged as having high risk of bias	Both patient blinding and assessor blinding were judged as having unclear risk of bias.
Study described as double blind	“Double blind” is the description in the study related to “blindness.” Also, it should be stated that neither the person doing the assessments nor the study participants could identify the intervention being assessed.	Not described as double blind.	There is insufficient information to permit a judgment.
blinding of patients, assessors, and caregivers concurrently	Both patient blinding and assessor blinding were judged as having low risk of bias. Also, care-providers are blinded.	Both patient blinding and assessor blinding were judged as having high risk of bias. Also, care-providers are not blinded.	Both patient blinding and assessor blinding were judged as having unclear risk of bias. Also, care-providers are judged as “unclear”.
The method of blinding was appropriate	The authors use the blinding method appropriately. Blinding of participants/patients is a “must” when outcomes are subjective or self-reported. When outcomes are measured by an assessor, the assessors should be blinded to group allocation. Also, score “completely done” when it is unlikely that the blinding could have been broken and the nonblinding of others is unlikely to introduce bias. No blinding, but the review authors judge that the outcome and the outcome measurement are not likely to be influenced by lack of blinding. Objectives automatized outcomes coming from databases or hospital register office.	There is no blinding or incomplete blinding is performed, and the outcome or outcome measurement is likely to be influenced by lack of blinding.	There is insufficient information to permit a judgment.

We scored each item following the definitions and methods for each criterion in the quality assessment tools that were found to be valid and most commonly used in health research [7, 31–38]. We established our evaluation based on the chosen primary outcome of analysis, and employed a 3-level ordinal scoring scheme comprised of “high, unclear, low” risk of bias [39] for two domains (patient blinding and assessor blinding) and “yes, no, unclear” [7] for the other five domains. See Table 1 for further details of the blinding-based criteria used in the study.

Moreover, we assessed whether each individual component of a trial (participants, assessors, care-providers, statisticians, or investigators) would be blinded to study measurements: random assignment, hypothesis, details of interventions, outcome measures, and outcome analysis.

### Data analysis

To describe the blinding in the RCTs selected, we conducted descriptive analyses including proportions and percentages of study elements. To examine whether dental RCTs with adequate blinding reported different treatment ES estimates than trials with lack of blinding, we conducted a two-level analysis using a meta-meta-analytic approach with a random-effects model following guidelines established by Sterne et al. [40]. This type of analysis is reported to be the most effective to address our research question, given that the methodological approach used for our meta-epidemiological analysis takes into account heterogeneity between randomized trials, within meta-analyses in a first step, and among meta-analyses in a second step [41, 42]. We obtained raw data for each trial from each meta-analysis and cross-checked the numbers with the data reported in the primary trial.

For the “within meta-analyses level” (first level analysis), we obtained a standardized treatment ES estimate for the primary outcome of each randomized trial, as outlined by Cohen [43]. A negative treatment ES estimate entailed a favourable effect of the tested intervention. We obtained data from each selected randomized trial and meta-analysis. We considered a trial if it was included in more than one meta-analysis, only once (from the meta-analysis with the fewer number of trials). We divided included trials, for each meta-analysis and each randomized trial component, into two groups according to the relevant quality criterion (e.g., participant blinding, assessor blinding, care-provider blinding)—those that adequately addressed the criterion and those that did not (“no” or “unclear”). We calculated two treatment ES estimates for each meta-analysis: the first corresponded to the pooled treatment ES estimate from trials including the characteristic of interest (e.g., patient blinding) and the second corresponded to the pooled treatment ES estimate from trials where the characteristic of interest (e.g., no or unclear patient blinding) was not met. We conducted inverse-variance random-effects meta-analysis to derive pooled treatment ES estimates for each meta-analysis, and calculated the DerSimonian and Laird estimates of variance to determine heterogeneity between randomized trials [41]. Thus, for each meta-analysis, we used meta-regression approaches to derive the difference between pooled estimates from trials with and without the characteristic of interest, as well as its standard error. A negative difference in treatment ES estimate implied that trials with the blinding-based item yielded a more favourable treatment ES estimate for the tested intervention.

For the “among meta-analyses level” (second level analysis), we pooled findings of the previous analysis (combined differences from all meta-analyses) to describe the effect of each trial’s component across all meta-analyses. We combined differences in treatment ES estimates at this stage using inverse-variance random-effects meta-analysis [41] to account for between-meta analysis heterogeneity, and calculated the DerSimonian and Laird estimates of variance to determine heterogeneity between meta-analyses [41]. All *p*-values were two-sided.

To examine whether specific characteristics modify the associations between blinding and the treatment ES estimate, we stratified the analyses with interaction tests based on Z scores according to the following factors: type of outcome (objective vs. subjective), dental speciality (periodontal vs. other interventions, or dental public health vs. other interventions), magnitude of the treatment ES estimate (small, if > − 0.5 vs. large, if ≤ − 0.5), and heterogeneity of the meta-analysis (low if τ^2^ < 0.06 vs. high if τ^2^ ≥ 0.06; the cut-off of τ^2^ = 0.06 roughly amounts to a difference between the largest and the smallest treatment ES estimate, where the smallest treatment ES estimate = 1). We performed all analyses using STATA statistical software version 14 (College Station, TX: StataCorp LP). The analysis was conducted by the principal investigator who was trained, and supervised by a team member with extensive experience in analyses of meta-epidemiological studies.

We calculated the sample size according to recommendations in Hempel et al. [44] and Berkman et al. [24]. From previous meta-epidemiological investigations [30, 45, 46], we anticipated obtaining a difference in treatment ES estimates of at least 0.15 (SE = 0.087) between trials with and without quality criteria [45]. This magnitude of difference in treatment ES estimates has been claimed to resemble nearly 1/4 to 1/2 of classic treatment ES estimates for interventions in fields similar to the field of dentistry [45]. Accordingly, we planned a sample size of nearly 500 randomized trials included in 60 systematic reviews to demonstrate such a meaningful difference. This is approximately two to three times the number of trials included in previously published meta-epidemiological investigations [18, 45, 47].

## Results

### Characteristics of selected systematic reviews and included randomized trials

The updated database of dental, oral, and craniofacial systematic reviews [45] included 1408 records (published between 1991 and 2014) of which 152 systematic reviews with meta-analyses were judged to be potentially relevant; of these, 64 (32 Cochrane and 32 non-Cochrane reviews) satisfied the eligibility criteria for the present report. The complete list of excluded reviews is available upon request.

Overall, the chosen meta-analyses were published between 2002 and 2014 (median year of publication: 2010; interquartile range [IQR] 2006–2012), while the median number of trials included in the meta-analyses was six (IQR 6–10). A total of 540 trials analyzing 137,957 patients were considered for this study [48, 49]. The meta-analyses examined a therapeutic intervention related to the fields of periodontics (36 reviews; 271 trials), dental public health and pediatric dentistry (10 reviews; 145 trials), oral medicine and pathology (11 reviews; 80 trials), oral and maxillofacial surgery (4 reviews; 26 trials), orthodontics and dentofacial orthopedics (2 reviews; 12 trials), and restorative dentistry (1 review; 6 trials). Approximately one-fifth of the trials were multicenter trials, nearly one-third of the trials placebo-controlled (*n* = 204; 37.8%), and two-thirds of the trials examined were nondrug (*n* = 359; 66.5%) or nonsurgical (*n* = 370; 68.5%) interventions. The majority of trials used parallel design (*n* = 372; 68.9%), and one-quarter used split-mouth design (*n* = 126; 23.3%). Additional file 2: Appendix 1 provides further details on characteristics of the chosen meta-analyses.

### Blinding in dental randomized trials

Blinding of patients was judged as adequate (low risk of bias) in 71.5% (*n* = 386) of the trials, and blinding of the outcome assessment was judged as adequate (low risk of bias) in 59.4% of the trials. Blinding of both patients and assessors was judged as adequate in 72.8% of trials (*n* = 273), and 76.5% (*n* = 117) of trials were assessed as adequate with respect to blinding of patients, assessors, and care-providers. Blinding of the assessor was reported in 59.4% of trials (*n* = 321), while blinding of patients was unclear/not reported in nearly half of trials (*n* = 279; 51.7%). Two-thirds of trials were not described as double-blind (*n* = 358; 66.3%). The method of blinding was appropriate in 53% of trials (*n* = 286), while blinding of the principal investigator and statistician was unclear/not reported in the vast majority of trials. Tables 2 and 3 provide details of the blinding of individual components (participants, assessors, principal investigators, care-providers, and statisticians), and the level of blinding (random assignment, hypothesis, details of intervention, and data analysis) in RCTs of oral health interventions.

**Table 2:** Blinding in randomized trials of oral health interventions (*N* = 540)

Domain	Risk of Bias Assessment, N (%)		
	Low Risk	High Risk	Unclear Risk
Blinding of patients/participants	386 (71.5)	7 (1.3)	147 (27.2)
Blinding of assessors	321 (59.4)	16 (3.0)	203 (37.6)
Blinding of both patients and assessors^a^	273 (72.8)	7 (1.9)	95 (25.3)
Blinding of patients, assessors, and care-providers concurrently^b^	117 (76.5)	7 (4.6)	29 (19.0)
Item	Quality Assessment, N (%)		
	Yes	No	Unclear/Not reported
Study described as double-blind	181 (33.5)	358 (66.3)	1 (0.2)
Blinding of assessors	321 (59.4)	16 (3.0)	203 (37.6)
Blinding of patients	192 (35.6)	69 (12.8)	279 (51.7)
Blinding of therapists/care-providers	134 (24.8)	356 (65.9)	50 (9.3)
Blinding of principal investigator	33 (6.1)	10 (1.9)	497 (92.0)
Blinding of data analyst	9 (1.7)	3 (0.6)	528 (97.8)
Method of blinding appropriate	286 (53)	17 (3.1)	237 (43.9)

^a^Does not equal 100% for overall, as the item was not applicable in 165 trials

^b^Does not equal 100% for overall, as the item was not applicable in 387 trials

**Table 3:** Type of blinding in randomized trials of oral health interventions (*N* = 540); N (%)

Component	Random allocation			Hypothesis			Details of intervention			Outcome assessment			Data analysis		
	Yes	No	Unclear/NR	Yes	No	Unclear/NR	Yes	No	Unclear/NR	Yes	No	Unclear/NR	Yes	No	Unclear/NR
Participants	194 (35.93)	70 (12.96)	276 (51.11)	1 (0.19)	12 (2.22)	527 (97.59)	2 (0.37)	221 (40.93)	317 (58.70)	0 (0.0)	71 (13.15)	469 (86.85)	0 (0.0)	0 (0.0)	540 (100.0)
Assessors	322 (59.63)	15 (2.78)	203 (37.59)	1 (0.19)	11 (2.04)	528 (97.78)	8 (1.48)	37 (6.85)	495 (91.67)	NA	NA	NA	0 (0.0)	0 (0.0)	540 (100.0)
Principal Investigator	32 (5.93)	10 (1.85)	498 (92.22)	0 (0.0)	0 (0.0)	540 (100.00)	0 (0.0)	0 (0.0)	540 (100.0)	1 (0.19)	2 (0.37)	533 (99.44)	0 (0.0)	0 (0.0)	540 (100.0)
Care-providers	136 (25.19)	351 (65.00)	53 (9.81)	2 (0.37)	10 (1.85)	528 (97.78)	0 (0.0)	0 (0.0)	540 (100.0)	1 (0.19)	16 (2.96)	523 (96.85)	0 (0.0)	0 (0.0)	540 (100.0)
Statisticians	9 (1.67)	3 (0.56)	528 (97.78)	1 (0.19)	0 (0.0)	539 (99.81)	0 (0.0)	0 (0.0)	540 (100.0)	0 (0.0)	0 (0.0)	540 (100.0)	NA	NA	NA

*NA* not applicable, *NR* not reported

### Impact of patient blinding on treatment effect size estimate

**Fig. 1:**
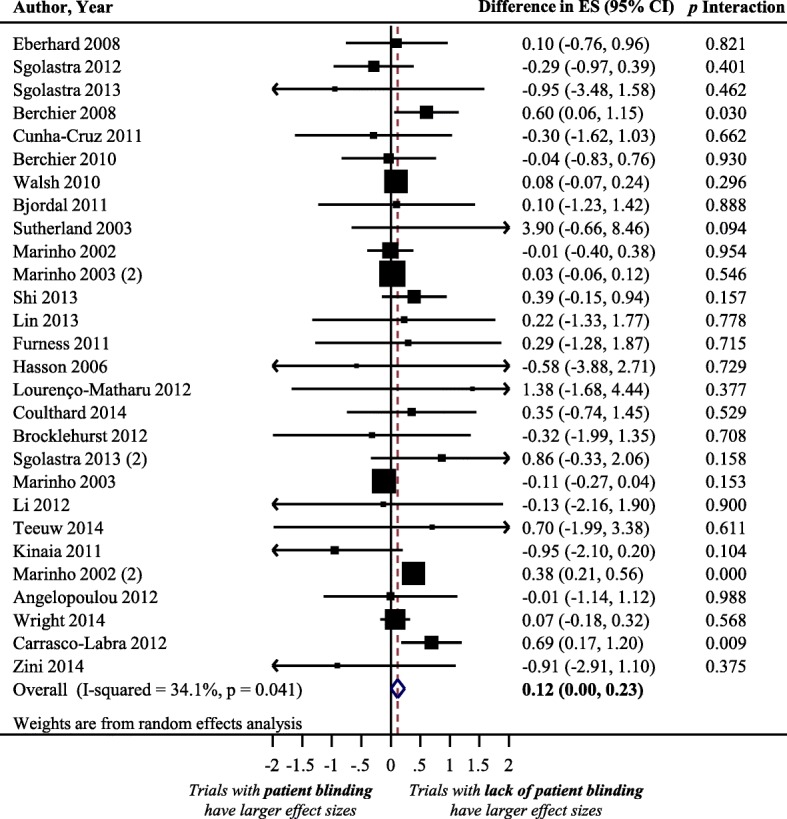
Difference in treatment ES estimates between trials with presence and lack of patient blinding. A positive value (more than zero) across meta-analyses indicates that treatment ES estimates are larger in trials that lack patient blinding compared to trials with adequate patient blinding. Diamond, difference in treatment ES estimate between trial components across all meta-analyses; square, proportional to weight used in meta-meta-analysis; horizontal arrow/line, a 95% confidence interval; solid vertical line, line of no difference in treatment ES estimate

**Fig. 2:**
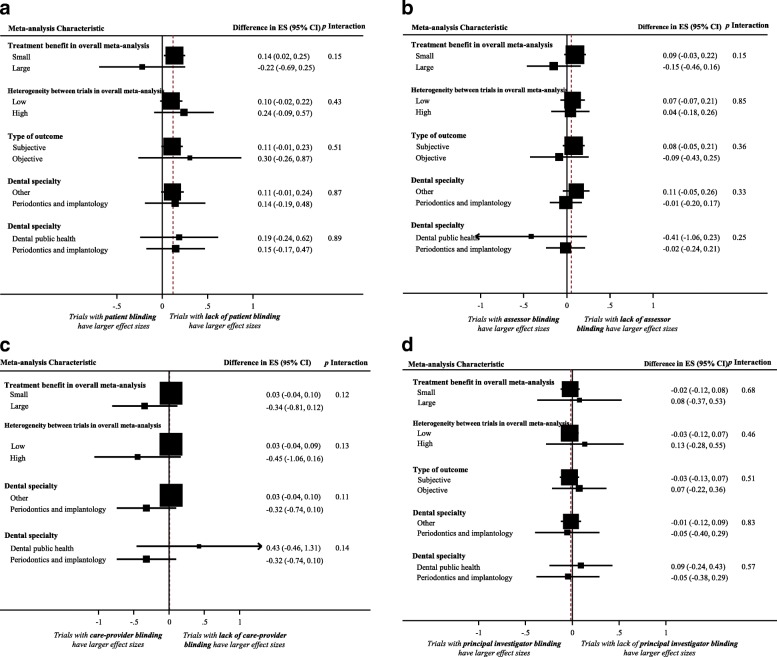
Difference in treatment ES estimates, stratified by meta-analyses characteristics, between: (**a**) trials with presence and lack of patient blinding; (**b**) trials with presence and lack of assessor blinding; (**c**) trials with presence and lack of care-provider blinding; (**d**) trials with and without principal-investigator blinding. Square, proportional to weight used in meta-meta-analysis; horizontal arrow/line, a 95% confidence interval; solid vertical line, line of no difference in treatment ES estimate

Figure 1 displays a forest plot of the difference in treatment ES estimates between trials with the presence and lack of patient blinding. Twenty-eight meta-analyses, including 275 trials that analyzed 109,753 patients, provided information for this meta-epidemiological analysis. Results of the analysis showed that trials with inadequate patient blinding had significantly larger treatment ES estimates (difference = 0.12, 95% confidence interval 0.00 to 0.23, *p* = 0.046). However, the impact of patient blinding on treatment ES estimates stratified by other characteristics of meta-analyses (heterogeneity of meta-analysis, type of outcome, and dental speciality) was not statistically significant for any of the characteristics (see Fig. 2a**)**.

### Impact of assessor blinding on treatment effect size estimate

**Fig. 3:**
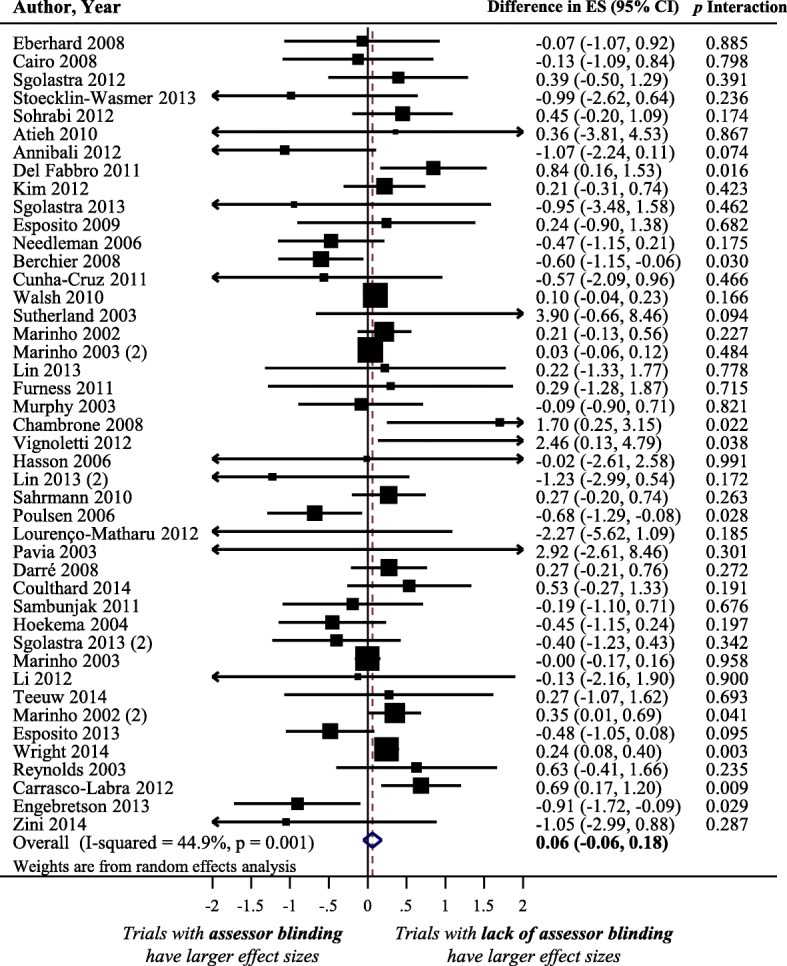
Difference in treatment ES estimate between trials with presence and lack of assessor blinding. A positive value (more than zero) across meta-analyses indicates that lack of assessor blinding inflates the treatment ES estimate when compared with trials with adequate assessor blinding. Diamond, difference in treatment ES estimate between trial components across all meta-analyses; square, proportional to weight used in meta-meta-analysis; horizontal arrow/line, a 95% confidence interval; solid vertical line, line of no difference in treatment ES estimate

Figure 3 displays a forest plot of the difference in treatment ES estimates between trials with a presence and a lack of assessor blinding. Forty-four meta-analyses, including 408 trials that analyzed 119,282 patients, provided information for this meta-epidemiological analysis. Although assessor blinding was not associated with a statistically significant difference in treatment ES, trials with lack of assessor blinding tended to inflate treatment ES estimates when compared with trials with a presence of assessor blinding (difference = 0.06, 95% confidence interval − 0.06 to 0.18, *p* = 0.316). The results of the stratified analyses show that none of the meta-analyses characteristics had a statistically significant interaction with the treatment ES estimate (see Fig. 2b**)**.

### Impact of care-provider blinding on treatment effect size estimate

**Fig. 4:**
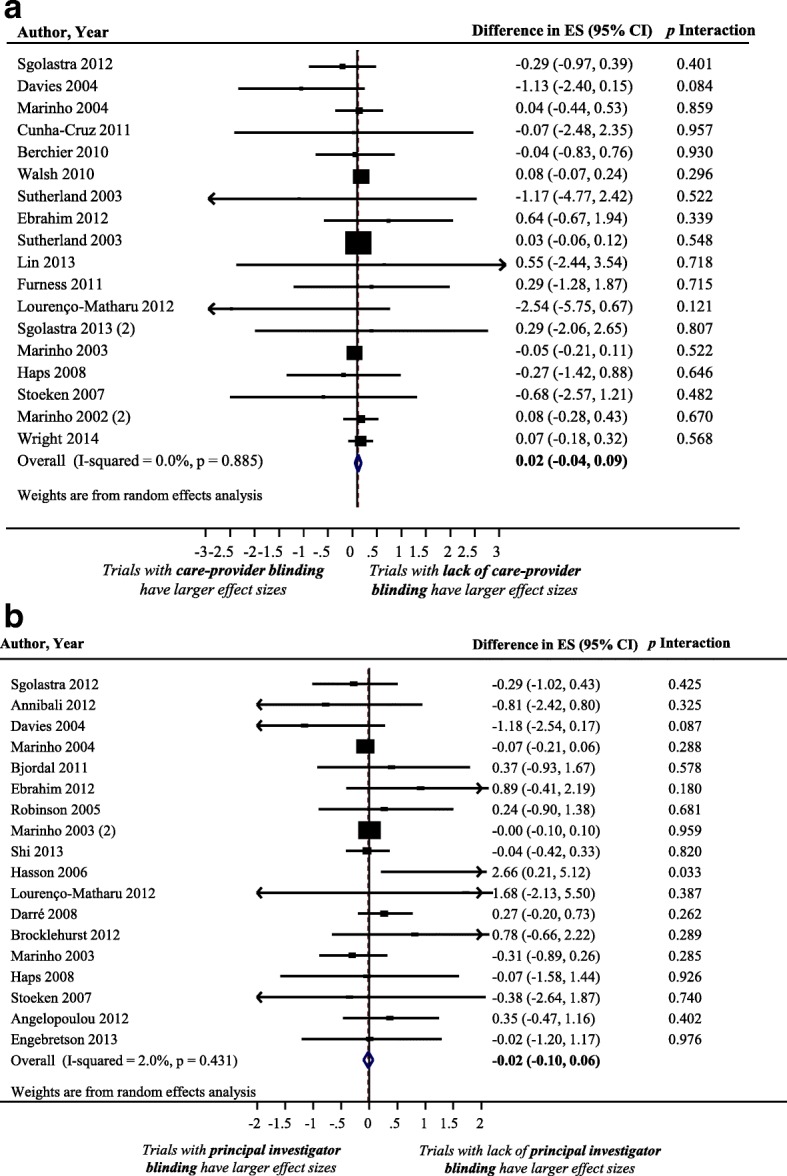
Difference in treatment ES estimate between: (**a**) trials with presence and lack of care-provider blinding (a positive value across meta-analyses indicates that the lack of care-provider blinding inflates the treatment ES estimate when compared with trials with adequate care-provider blinding); (**b**) trials with presence and lack of principal-investigator blinding (a positive value across meta-analyses indicates that the lack of principal-investigator blinding inflates the treatment ES estimate when compared with trials with adequate principal investigator blinding). Diamond, difference in treatment ES estimate between trial components across all meta-analyses; square, proportional to weight used in meta-meta-analysis; horizontal arrow/line, a 95% confidence interval; solid vertical line, line of no difference in treatment ES estimate

Figure 4a displays a forest plot of the difference in treatment ES estimates between randomized trials with a presence and a lack of care-provider blinding. Eighteen meta-analyses, including 408 trials that analyzed 109,383 patients, provided information for this meta-epidemiological analysis. Care-provider blinding was not associated with a statistically significant difference in treatment ES estimates (difference = 0.02, 95% confidence interval − 0.04 to 0.09, *p* = 0.509). The results of the stratified analyses show that none of the meta-analyses characteristics had a statistically significant interaction with the treatment ES (see Fig. 2c**)**.

### Impact of principal-investigator blinding on treatment effect size estimate

Figure 4b displays a forest plot of the difference in treatment ES estimates between randomized trials with a presence and a lack of principal-investigator blinding. Eighteen meta-analyses, including 162 trials that analyzed 59,757 patients, provided information for this meta-epidemiological analysis. Principal-investigator blinding was not associated with a statistically significant difference in treatment ES estimates (difference = − 0.02, 95% confidence interval − 0.10 to 0.06, *p* = 0.641). Results of stratified analyses show that none of the meta-analyses characteristics had a statistically significant interaction with the treatment ES (see Fig. 2d).

### Impact of data-analyst blinding on treatment effect size estimate

Due to the small number of trials with adequate blinding of the data-analyst, meta-epidemiological analysis of the data could not be performed for this criterion.

### Impact of describing a trial as “double-blind” on treatment effect size estimate

**Fig. 5:**
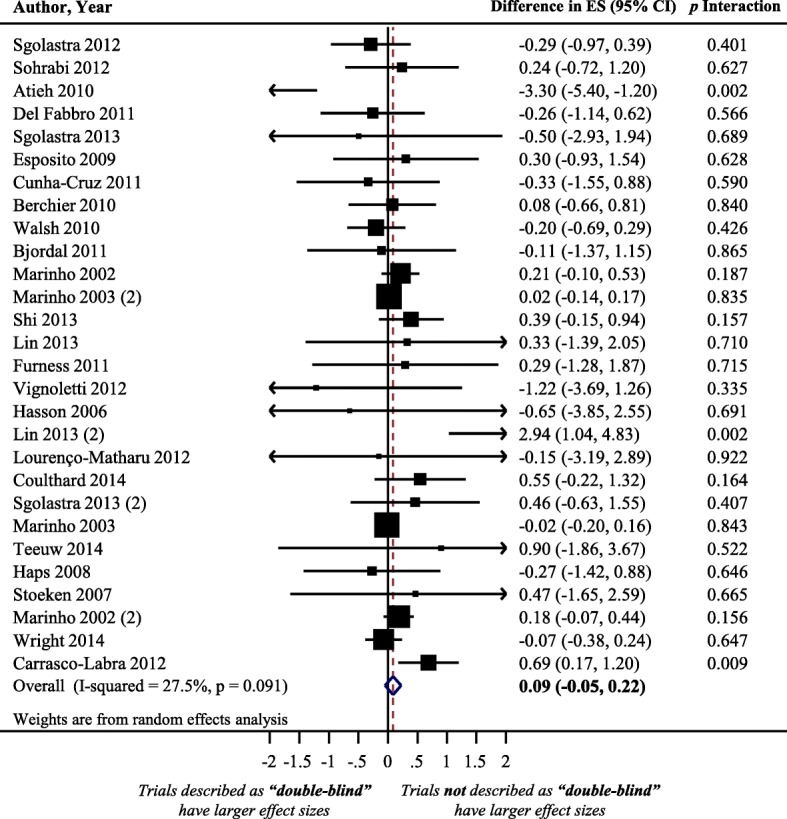
Difference in treatment ES estimate between trials with presence and lack of “double-blinded” description. A positive value (more than zero) across meta-analyses indicates that trials not described as “double-blinded” inflate the treatment ES estimate when compared with trials described as “double blinded”. Diamond, difference in treatment ES estimate between trial components across all meta-analyses; square, proportional to weight used in meta-meta-analysis; horizontal arrow/line, a 95% confidence interval; solid vertical line, line of no difference in treatment ES estimate

**Fig. 6:**
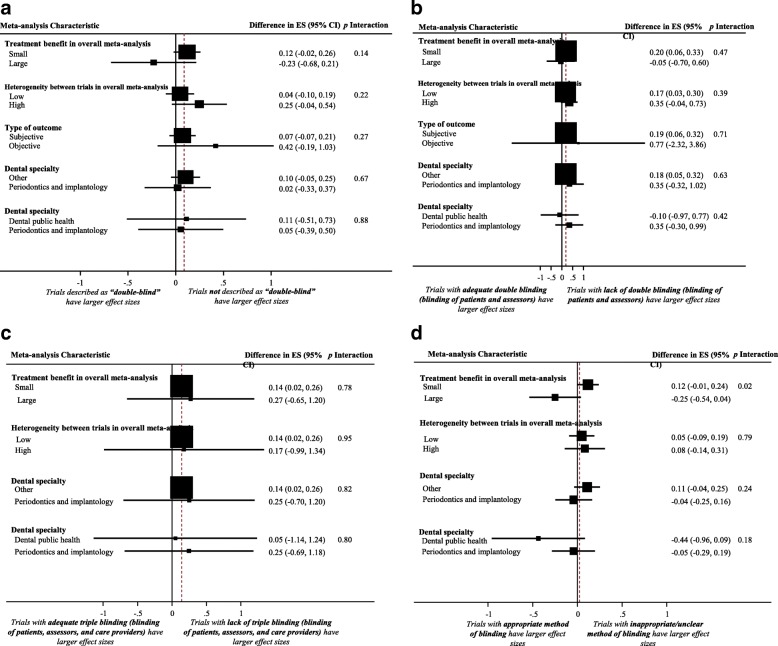
Difference in treatment ES estimates, stratified by meta-analyses characteristics, between: (**a**) trials with presence and lack of “double-blinded” description; (**b**) trials with and without blinding of both patients and assessors; (**c**) trials with and without blinding of patients, assessors, and care providers concurrently; (**d**) trials with and without appropriate method of blinding. Square, proportional to weight used in meta-meta-analysis; horizontal arrow/line, a 95% confidence interval; solid vertical line, line of no difference in treatment ES estimate

Figure 5 displays a forest plot of the difference in treatment ESs between randomized trials with and without reporting “double blinding.” Twenty-eight meta-analyses, including 294 trials that analyzed 111,052 patients, provided information for this meta-epidemiological analysis. Trials not described as double-blind tended to exaggerate treatment ES estimate compared to trials described as double-blind. However, differences were not statistically significant (difference = 0.09, 95% confidence interval − 0.05 to 0.22, *p* = 0.203). The results of stratified analyses showed that none of the meta-analyses characteristics had a statistically significant interaction with the treatment ES (see Fig. 6a**)**.

### Impact of blinding of both patients and assessors on treatment effect size estimate

**Fig. 7:**
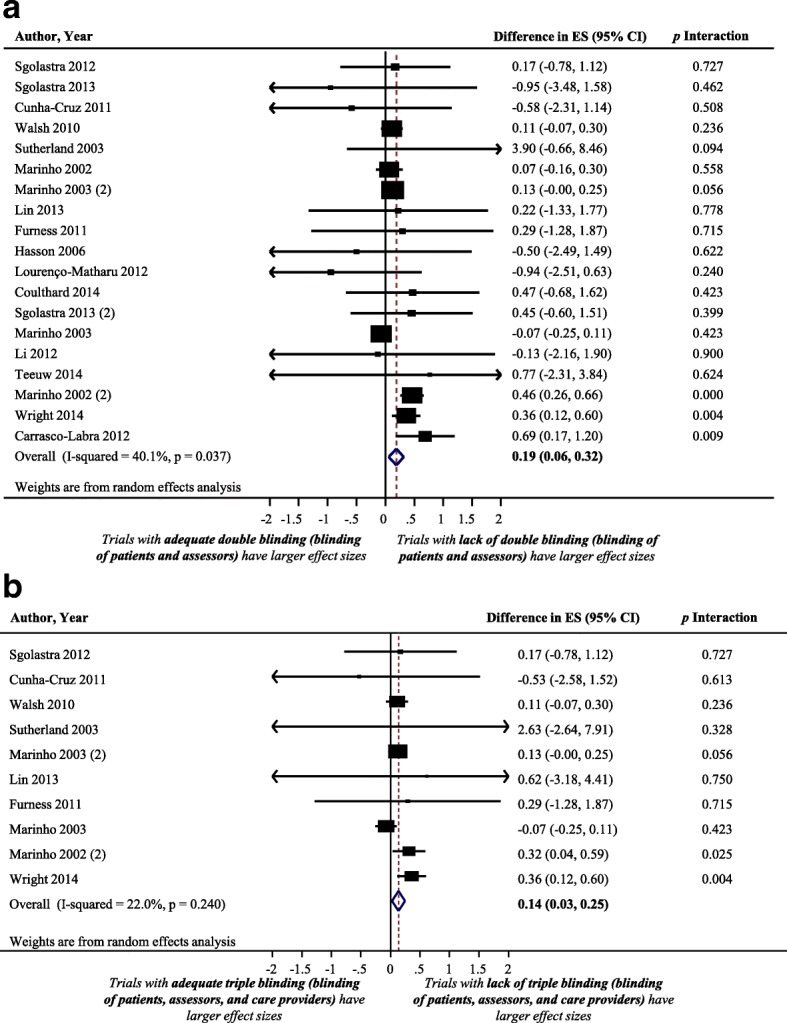
Difference in treatment ES estimate between: (**a**) trials with and without blinding of both patients and assessors (a positive value, more than zero, across meta-analyses indicates that lack of blinding of both patients and assessors inflates the treatment ES estimate when compared with trials with adequate blinding of patients and assessors); (**b**) trials with and without blinding of patients, assessors, and care providers (a positive value, more than zero, across meta-analyses indicates that lack of blinding of patients, assessors, and care providers inflates the treatment ES estimate when compared with trials adequately blinded in the three components). Diamond, difference in treatment ES estimate between trial components across all meta-analyses; square, proportional to weight used in meta-meta-analysis; horizontal arrow/line, a 95% confidence interval; solid vertical line, line of no difference in treatment ES estimate

Figure 7a shows a forest plot of the difference in treatment ES estimate in randomized trials with and without blinding of both patients and assessors. Nineteen meta-analyses, including 224 trials that analyzed 106,716 patients, provided information for this meta-epidemiological analysis. Meta-epidemiological results showed a statistically significant difference between the treatment ES estimate in RCTs that implemented patient and assessor blinding concurrently (difference = 0.19, 95% confidence interval 0.06 to 0.32, *p* = 0.004) and the treatment ES estimate in randomized trials that did not employ blinding of both patients and assessors. However, the impact of blinding of both patients and assessors on treatment ES estimate stratified by examined characteristics of meta-analyses was not statistically significant for any of the characteristics (see Fig. 6b**)**.

### Impact of blinding of patients, assessors, and care-providers concurrently on treatment effect size estimate

Figure 7b shows a forest plot of the difference in treatment ES estimate between randomized trials with the presence and lack of blinding of patients, assessors, and care-providers concurrently. Ten meta-analyses, including 151 trials that analyzed 99,293 patients, provided information for this meta-epidemiological analysis. Results of the analysis showed that trials that did not implement patient, assessor, and care-provider blinding had significantly larger treatment ESs (difference = 0.14, 95% confidence interval 0.03 to 0.25, *p* = 0.013) than trials that implemented blinding of those three components. However, results of the stratified analyses show that none of the examined meta-analyses characteristics had a statistically significant interaction with the treatment ES (see Fig. 6c**)**.

### Impact of using an appropriate method of blinding on treatment ES

**Fig. 8:**
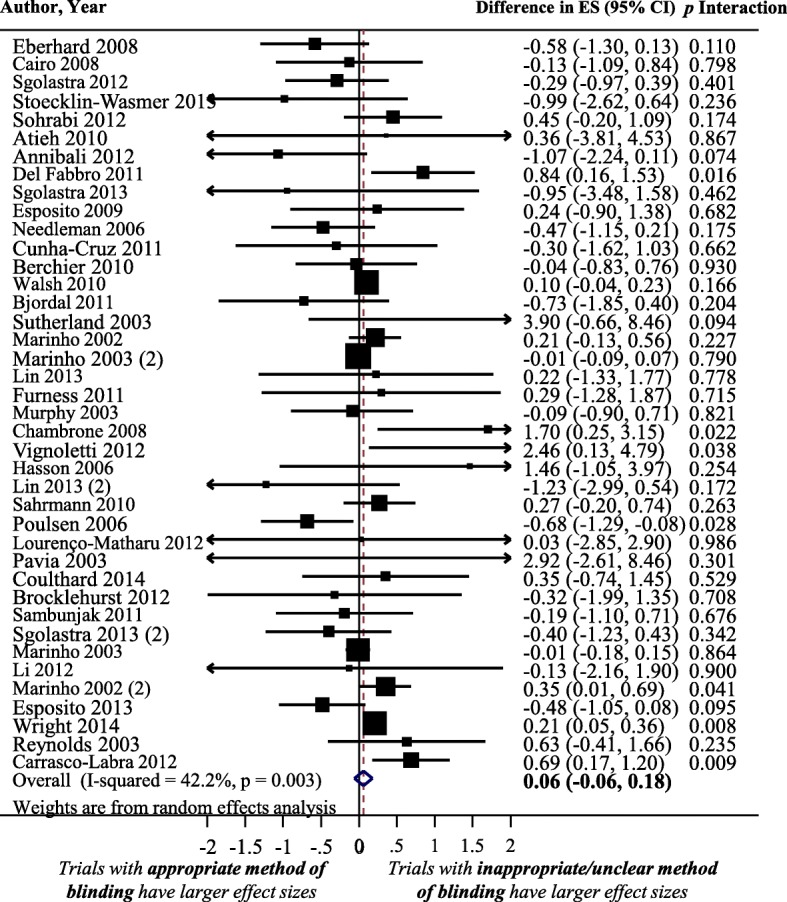
Difference in treatment ES estimate between trials with and appropriate method of blinding (a positive value, more than zero, across meta-analyses indicates that lack of an appropriate method of blinding inflates the treatment ES estimate). Diamond, difference in treatment ES estimate between trial components across all meta-analyses; square, proportional to weight used in meta-meta-analysis; horizontal arrow/line, a 95% confidence interval; solid vertical line, line of no difference in treatment ES estimate

Figure 8 shows a forest plot of the difference in treatment ES estimates between trials with the presence and lack of an appropriate method of blinding. Forty meta-analyses provided information for this meta-epidemiological analysis. Presence of an appropriate method of blinding was not associated with a statistically significant difference in treatment ES estimate, trials that lacked an appropriate method of blinding tended to inflate treatment ES estimates compared to trials with an appropriate method of blinding (difference = 0.06, 95% confidence interval − 0.06 to 0.18, *p* = 0.325). The results of the stratified analyses showed that differences in treatment ES estimates between trials with the presence or lack of appropriate blinding were significant (*p* < 0.02) in meta-analyses with a large treatment benefit in overall meta-analysis, but not in meta-analyses with a small treatment benefit. However, none of the other considered factors (heterogeneity of meta-analysis, type of outcome, and dental specialty) had a statistically significant interaction with the treatment ES estimate (see Fig. 6d).

## Discussion

Our investigation provides empirical evidence of the impact of bias associated with nine blinding-based criteria (related to patient, assessor, care-provider, and principal-investigator blinding) on the treatment ES estimate. This analysis is important to methodologists and researchers in dental, oral, and craniofacial research. To our knowledge, this study is the first meta-epidemiological study conducted in any medical or dental field that examines continuous outcomes of the impact of blinding of both patients and assessors and of patients, assessors, and care-providers on treatment ES estimates in randomized trials.

Our study shows significant differences in treatment ES estimates in oral health RCTs based on different types of blinding. For example, RCTs with lack of patient and assessor blinding had significantly larger treatment ES estimates compared to trials without lack of patient and/or assessor blinding. Patient blinding and assessor blinding were associated with inflated treatment ES estimates (significant at the level of patient blinding), while care-provider and principal-investigator blinding were not related to inflated treatment ES estimates. Interestingly, lack of blinding of both assessors and patients was found to be associated with the largest overestimation in treatment ES estimate (0.19). This measured magnitude of bias represents approximately 1/3 of common treatment ES estimates reported in oral health research [50], such as clinical outcomes in periodontology [46]. The fact that treatment ES estimates in oral health trials may have been biased due to lack of blinding is concerning, as clinical decision making related to recommended dental treatments and modalities may therefore not be based on valid findings.

The stratified analyses showed that the extent of bias associated with lack of blinding was not significantly associated with any other factor considered at the meta-analysis level. This agrees with a recent study conducted in the area of physical therapy, and is contrary to other meta-epidemiological studies [51], which showed that trials with subjective outcomes exaggerated treatment ES estimates compared to trials with objective outcomes. This could be due to having a small number of trials with objective outcomes in our study, or to differences between interventions in different medical disciplines.

Reports examining the impact of lack of blinding of patient, therapist, or assessor on treatment ES estimates were conducted in particular medical fields such as physical therapy [19], thrombosis and cardiovascular disease [15, 21], pediatrics [18], osteoarthritis [45], and low-back pain [16]. The studies reported inconsistent findings. The treatment ES estimate was smaller in trials that employed patient blinding [15] or assessor blinding [20, 21] in some studies, whereas in other studies the treatment ES estimate was smaller in trials that lacked patient [17] or assessor blinding [15]. However, an association between the treatment ES estimate and the presence or lack of blinding was not confirmed in some studies [16, 45]. Furthermore, while the definition of double blinding varied largely among the meta-epidemiological studies with respect to the level of blinding (patient, assessor, and care-provider blinding), a lack of double blinding was found to be associated with exaggerated treatment ES estimates in general [22, 23, 52]. The inconsistent findings might be due to the examination of different types of outcome, intervention, and population, to the implementation of different definitions of quality assessment, and to the application of various statistical and modeling approaches [24]. For example, Schulz et al. [52] applied a multiple logistic regression model to analyze data on binary outcomes from 250 trials included in 33 meta-analyses; the definition of double blinding was based on whether the trial’s conduct claimed to be double-blinded. Egger et al. [22] defined “double blinding” based on whether the trial was described as double-blind, or included at least assessor blinding; the study analyzed data from 304 trials included in 39 meta-analyses with binary outcomes in several medical fields (infectious diseases, neurology, among others).

Two recent studies [18, 19] that examined the association between lack of blinding of patient, therapist, or assessor, and treatment ES using continuous outcomes, also reported inconsistent findings. One study assessed the adequacy of patient and assessor blinding in 287 pediatric trials from 17 meta-analyses [18], and showed no significant difference in treatment ESs between studies, based on potential bias related to lack of blinding. Another study assessed 165 physical therapy trials included in 17 meta-analyses and found that trials with a lack of patient or assessor blinding tended to underestimate treatment ES estimate when compared with trials with appropriate blinding (although, the differences were not statistically significant) [19]. It should be noted that in both studies, lack of significant results might be accounted for by the small number of trials, precision of the analyses performed, and/or examination of interventions where blinding is not crucial or fundamental (i.e., outcomes are objective or automated with no assessor involvement).

Because the concept of blinding is implemented at multiple levels of a trial (e.g., patients, assessors, care providers, data analysts, investigators), there is confusion when describing the level of blinding implemented. For example, “double blinding” or “triple blinding” may refer to blinding at any two or three of the previous levels. Failure to clearly report the levels that such terms refer to leads to confusion. Investigators of RCTs conducted in the field of dentistry need to implement and clearly report blinding of patients, assessors, care providers, data analysts, and other personnel when applicable, and explicitly report on mechanisms used to achieve and assure successful blinding, as recommended by the Consolidated Standards of Reporting Trials (CONSORT) statement. In addition, investigators of RCTs should state the levels (e.g., patients, assessors, care providers) and components (e.g. allocation, outcomes assessed, details of interventions) they are referring to when they describe blinding of a trial. In addition, they should avoid using the terms “double” or “triple” blind trial when reporting trial findings, and report who was blinded and to what components blinding was achieved, so the reader can evaluate potential associated bias. As well, editors and peer reviewers of dental journals should require authors of randomized trials to adhere to the CONSORT guidelines and insist on adequate conduct and reporting of blinding in submitted randomized trials.

When we examined the association between double blinding and treatment ES, we performed the analysis on two different criteria: reporting of “double blinding” as a term in a trial, and actual conduct of blinding of both assessors and patients. Haahr and Hróbjartsson [53], who examined a random sample of RCTs from the Cochrane Central Register of Controlled Trials, suggested that it is incorrect to assume blinding of a trial participant based only on the term “double blind.” The study found that blinding of patients, care providers, and assessors was clearly described in only three (2%) of 200 blinded RCTs, while 56% of trials failed to describe blinding status of any individual involved in a trial. That study concluded that either patients, care providers, or assessors were not blinded in one of five “double blind” RCTs. Another trial study [54] showed that adequate reporting of blinding was common in some medical journals, and that inadequate reporting of blinding does not necessarily entail a lack of actual blinding. For example, it was reported that RCT authors frequently use blinding, although they fail to describe its methods. For instance, authors of RCTs failed to report the blinding status of patients in 26% of trials, and patients were actually blinded in 20% of trials in which patients were not reported to be blinded. Similar results were found in a recent study by Kahan et al. [55], who reported that blinding of outcome assessors is uncommonly used and inadequately reported in a cohort of 258 trials published in four high-impact medical journals.

An implication that can be drawn from our meta-epidemiological work is that authors of systematic reviews of oral health interventions should consider excluding dental RCTs with lack of blinding from meta-analyses, or at least perform sensitivity analyses on included trials based on the adequacy of blinding. In all instances, authors should consider the likely level of bias associated with reported (or unreported) blinding status when interpreting the findings of a quantitative analysis.

The above-mentioned implications should be considered with caution, particularly in oral health trials involving surgical or device interventions (such as orthodontic trials) where patient blinding is not feasible; in this case, informing patients with details of the intervention is required, and sometimes ethically compulsory. While these RCTs are prone to biases, particularly when the RCTs examine self-reported outcomes, implementation of blinding in the conduct of these trials is often unacceptable for ethical and practical reasons. For example, in the case of trials comparing surgical interventions to nonsurgical interventions (e.g., comparison of surgical removal of wisdom teeth versus retention or conservative management), patients and surgeons cannot be blinded. However, trialists may consider using “expertise-based” trial design, whereby patients are allocated to multiple surgeons and each surgeon performs a single treatment [56]. While this design helps to minimize performance bias related to surgeon blinding, it does not ensure patient blinding [57]. Furthermore, in trials where patients cannot be blinded (e.g., comparison of manual versus electric toothbrushing), trialists may consider using objective outcomes that have established validity and reliability [56] or blind patients to trial’s hypothesis. When blinding is feasible, trialists should consider blinding as many trial components (participants, assessors, care-providers, statisticians, investigators) as ethically and practically possible.

Based on this evidence, investigators of systematic reviews conducted in dental, oral, and craniofacial trials should perform sensitivity analyses based on the adequacy of blinding in included trials. The potential impact of blinding on bias in treatment ES suggests that dental journal editors and reviewers should insist on adequate blinding (when feasible) with respect to trial conduct and reporting, in published trials’ reports.

### Strengths and limitations of the study

This meta-epidemiological study provides an empirical analysis of the association between treatment ES estimates and bias, in the domain of oral health research. The study has several limitations.

First, we examined published studies only (bias was based on reported methodological characteristics), and did not evaluate actual conduct of the RCTs. Accordingly, data extraction and analyses were based on information given by authors in published reports. This approach, although widely used, limits the identification of actual bias if trial authors do not adequately report study elements.

Second, while there are many ways for an RCT planned as blinded to become unblinded, [58] our study did not use specific mechanisms to look for evidence of unblinding, such as differential (across treatment groups) incidences of specific adverse events that would give away which patients received which interventions and large baseline imbalances indicative of the type of selection bias that may occur with unsuccessful allocation concealment [59, 60]. Also, our study did not look at how many RCTs reported a valid and reliable method of assessment of the success of blinding such as the Berger-Exner test of selection bias [58]. Accordingly, future RCTs should routinely conduct and report the results of a valid and reliable method of assessing the success of blinding (such as the Berger-Exner test) based on the extent to which any unblinding led to selection bias [61, 62].

Third, certain levels of heterogeneity are expected in any meta-epidemiological examination of the impact of bias on treatment ES estimates. Such studies analyse numerous entities (meta-analysis, trials, and participants) that have a distinct potential for heterogeneity [24]. By applying a cautious methodology to data collection and analysis in this study, and by assembling a large number of meta-analyses and trials, study power was increased and heterogeneity was reduced.

Fourth, because our study did not compare the same treatment with different degrees of blinding, the identified evidence could lead to the conclusion that trials of interventions where blinding is not feasible, such as surgery or devices, have in general higher treatment ES estimates. Future meta-epidemiological studies should further investigate the above-mentioned concept.

Finally, this study did not assess the likely effects of interactions with other design biases. Such an assessment would have to include a multivariate analysis with a larger number of meta-analyses and trials [17]. Future meta-epidemiological assembling of a greater number of meta-analyses and trials by synthesizing results from different disciplines and datasets should take other design biases into account.

## Conclusions

We found significant differences in treatment ESs between oral health RCTs based on lack of patient and assessor blinding. RCTs that lacked patient and assessor blinding had significantly larger treatment ES estimates. Treatment ES estimates were 0.19 and 0.14 larger in trials with lack of blinding of both patients and assessors and blinding of patients, assessors, and care-providers concurrently. No significant differences were identified in other blinding criteria. Future meta-epidemiological assembling of a greater number of meta-analyses and trials that takes other biases and different degrees of blinding into account is needed.

## Additional files

Additional file 1: **Appendix 2.** This file contains search strategy used in the study. (DOCX 44 kb)

Additional file 2: **Appendix 1.** This file contains details of the meta-analyses included in the study. (DOCX 131 kb)

## Abbreviations

ES: Effect size
RCT: Randomized controlled trial
