# Supplementary material for: Influence of blinding on treatment effect size estimate in randomized controlled trials of oral health interventions
Source: BMC Med Res Methodol. 2018 May 18;18:42. doi: 10.1186/s12874-018-0491-0 (PMC5960173; doi:10.1186/s12874-018-0491-0)
Supplement: Supplementary file 1 — Appendix 2. This file contains search strategy used in the study. (DOCX 44 kb) [file 12874_2018_491_MOESM1_ESM.docx]

| **Appendix 1. Details of the meta-analyses included in the study** | | | | | | |
| --- | --- | --- | --- | --- | --- | --- |
| **Title of Meta-Analysis** | **Author and Year** | **Primary Dental Specialty** | **Outcome** | **Outcome Type** | **Comparison** | **No. of Trials** |
| Full-mouth disinfection for the treatment of adult chronic periodontitis | Eberhard 2008 [[1](#_ENREF_1)] | Periodontics | Bleeding on probing | Subjective | Full-mouth scaling vs. control | 5 |
| Treatment of gingival recession with coronally advanced flap procedures | Cairo 2008 [[2](#_ENREF_2)] | Periodontics | Gingival recession | Subjective | Coronally advanced flap plus enamel matrix derivative vs. coronally advanced flap | 5 |
| Effectiveness of systemic amoxicillin /metronidazole as an adjunctive therapy to full-mouth scaling and root planing in the treatment of aggressive periodontitis | Sgolastra 2012 [[3](#_ENREF_3)] | Periodontics | Clinical attachment levels | Subjective | Full-mouth scaling plus combined amoxicillin-metronidazole vs. full-mouth scaling | 6 |
| Absorbable collagen membranes for periodontal regeneration | Stoecklin 2013 [[4](#_ENREF_4)] | Periodontics | Clinical attachment levels | Subjective | Collagen membranes vs. control | 11 |
| An evaluation of bioactive glass in the treatment of periodontal defects | Sohrabi 2012 [[5](#_ENREF_5)] | Periodontics | Clinical attachment levels | Subjective | Bioactive glass vs. control | 14 |
| Platform switching for marginal bone preservation around dental implants | Atieh 2010 [[6](#_ENREF_6)] | Implantology | Marginal bone level | Subjective | Platform switch vs. platform match | 5 |
| Peri-implant marginal bone level | Annibali 2012 [[7](#_ENREF_7)] | Implantology | Marginal bone level | Subjective | Platform switch vs. platform match | 5 |
| Is platelet concentrate advantageous for the surgical treatment of periodontal diseases? | Del Fabbro 2011 [[8](#_ENREF_8)] | Periodontics | Clinical attachment levels | Subjective | Platelet-rich plasma vs. control | 10 |
| The effectiveness of a toothpaste containing triclosan and polyvinyl-methyl ether maleic acid copolymer in improving plaque control and gingival health | Davies 2004 [[9](#_ENREF_9)] | Periodontics | Gingival index | Subjective | Toothpaste vs. control | 6 |
| Scaling and root planing treatment for periodontitis to reduce preterm birth and low birth weight | Kim 2012 [[10](#_ENREF_10)] | Periodontics | Birth weight | Objective | Periodontal treatment vs. control | 6 |
| Lasers for the treatment of dentin hypersensitivity | Sgolastra 2013 [[11](#_ENREF_11)] | Oral medicine and pathology | Pain | Subjective | Laser vs. placebo | 13 |
| Combinations of topical fluoride (toothpastes, mouth rinses, gels, varnishes) versus single topical fluoride for preventing dental caries in children and adolescents | Marinho 2004 [[12](#_ENREF_12)] | Dental public health and pediatric dentistry | The decayed, missing, and filled surfaces (DMFS) index | Subjective | Fluoride toothpaste plus mouth rinse (or gel) vs. fluoride toothpaste | 6 |
| Enamel matrix derivative (Emdogain®) for periodontal tissue regeneration in intrabony defects | Esposito 2009 [[13](#_ENREF_13)] | Periodontics | Probing attachment level | Subjective | Emdogain vs. control | 9 |
| Guided tissue regeneration for periodontal infra-bony defects | Needleman 2006 [[14](#_ENREF_14)] | Periodontics | Attachment gain | Subjective | Guided tissue regeneration vs. control | 13 |
| The efficacy of dental floss in addition to a toothbrush on plaque and parameters of gingival inflammation | Berchier 2008 [[15](#_ENREF_15)] | Periodontics | Gingival index | Subjective | Floss plus toothbrushing vs. toothbrushing only | 5 |
| Dentin hypersensitivity and oxalates | Cunha-Cruz 2011 [[16](#_ENREF_16)] | Oral medicine and pathology | Dentin hypersensitivity | Subjective | Oxalate vs. placebo or no-treatment | 11 |
| The efficacy of 0.12% chlorhexidine mouth rinse compared with 0.2% on plaque accumulation and periodontal parameters | Berchier 2010 [[17](#_ENREF_17)] | Periodontics | Plaque index | Subjective | 0.12% vs. 0.2% chlorhexidine mouth rinse | 7 |
| Fluoride toothpastes of different concentrations for preventing dental caries in children and adolescents | Walsh 2010 [[18](#_ENREF_18)] | Dental public health and pediatric dentistry | The decayed, missing, and filled surfaces (DMFS) index | Subjective | Fluoride toothpaste vs. placebo | 19 |
| A systematic review with meta-analysis of the effect of low-level laser therapy (LLLT) in cancer therapy-induced oral mucositis | Bjordal 2011 [[19](#_ENREF_19)] | Oral medicine and pathology | Oral mucositis severity | Subjective | low-level laser therapy vs. placebo | 6 |
| Emergency management of acute apical periodontitis in the permanent dentition | Sutherland 2003 [[20](#_ENREF_20)] | Periodontics | Pain | Subjective | Treatment vs control | 5 |
| The effectiveness of splint therapy in patients with temporomandibular disorders | Ebrahim 2012 [[21](#_ENREF_21)] | Oral medicine and pathology | Pain | Subjective | Splint therapy vs. minimal/ no-treatment | 10 |
| Fluoride varnishes for preventing dental caries in children and adolescents | Marinho 2002 [[22](#_ENREF_22)] | Dental public health and pediatric dentistry | The decayed, missing, and filled surfaces (DMFS) index | Subjective | Fluoride varnish vs. placebo or no treatment | 7 |
| A review of the effects of stannous fluoride on gingivitis | Paraskevas 2006 [[23](#_ENREF_23)] | Periodontics | Gingival index | Subjective | SnF2 dentifrices vs. NaF | 6 |
| Manual versus powered toothbrushing for oral health | Robinson 2005 [[24](#_ENREF_24)] | Dental public health | Gingival index | Subjective | Side to side powered toothbrushes vs. manual toothbrushes | 8 |
| Fluoride toothpastes for preventing dental caries in children and adolescents | Marinho 2003 (2) [[25](#_ENREF_25)] | Dental public health and pediatric dentistry | The decayed, missing, and filled teeth (DMFT) index | Subjective | Fluoride toothpaste vs. placebo | 40 |
| Oral hygiene care for critically ill patients to prevent ventilator-associated pneumonia | Shi 2013 [[26](#_ENREF_26)] | Oral medicine and pathology | Duration of ventilation | Objective | Chlorhexidine vs. placebo or usual care | 6 |
| In-office treatment for dentin hypersensitivity | Lin 2013 [[27](#_ENREF_27)] | Oral medicine and pathology | Pain | Subjective | Physical occlusion (e.g., Pumice paste) vs. chemical occlusion (e.g., fluorides oxalates) | 6 |
| Triclosan/copolymer containing toothpastes for oral health | Riley 2013 [[28](#_ENREF_28)] | Periodontics | Plaque index | Subjective | Triclosan or copolymer vs. control | 10 |
| Interventions for the management of dry mouth: topical therapies | Furness 2011 [[29](#_ENREF_29)] | Oral medicine and pathology | Mouth dryness scale | Subjective | Saliva substitutes A vs. B | 6 |
| Guided tissue regeneration for the treatment of periodontal intrabony and furcation defects | Murphy 2003 [[30](#_ENREF_30)] | Periodontics | Clinical attachment levels | Subjective | Open flap debridement vs. guided tissue regeneration with barrier | 6 |
| Can subepithelial connective tissue grafts be considered the gold standard procedure in the treatment of Miller Class I and II recession-type defects? | Chambrone 2008 [[31](#_ENREF_31)] | Periodontics | Clinical attachment levels | Subjective | Guided tissue regeneration and membrane vs. subepithelial connective tissue graft | 7 |
| Surgical protocols for ridge preservation after tooth extraction | Vignoletti 2012 [[32](#_ENREF_32)] | Oral and maxillofacial surgery | Marginal bone level | Subjective | Surgical procedure vs. control | 7 |
| Home-based chemically-induced whitening of teeth in adults | Hasson 2006 [[33](#_ENREF_33)] | Restorative dentistry | Color | Subjective | Whitening product vs. whitening product - Colorimetric | 6 |
| The effect of flapless surgery on implant survival and marginal bone level | Lin 2013 [[34](#_ENREF_34)] | Implantology | Marginal bone level | Subjective | flapless vs. flap procedures | 5 |
| Impact of implant support for mandibular dentures on satisfaction, oral and general health-related quality of life | Emami 2009 [[35](#_ENREF_35)] | Implantology | Patient satisfaction | Subjective | Mandibular implant overdentures vs. conventional dentures | 6 |
| Systematic review on the effect of rinsing with povidone-iodine during nonsurgical periodontal therapy | Sahrmann 2010 [[36](#_ENREF_36)] | Periodontics | Periodontal probing depth | Subjective | Povidone-iodine rinsing vs. control | 5 |
| Potassium containing toothpastes for dentine hypersensitivity | Poulsen 2006 [[37](#_ENREF_37)] | Oral medicine and pathology | Tactile | Subjective | Potassium nitrate (no fluoride) vs. placebo (no potassium nitrate plus/- fluoride) | 5 |
| Sedation of children undergoing dental treatment | Lourenço-Matharu 2012 [[38](#_ENREF_38)] | Pediatric dentistry | Houpt/other behavioral score | Subjective | Sedatives vs. placebo | 6 |
| Meta-analysis of local tetracycline in treating chronic periodontitis | Pavia 2003 [[39](#_ENREF_39)] | Periodontics | Periodontal probing depth | Subjective | Tetracycline vs. placebo | 6 |
| Efficacy of periodontal treatment on glycaemic control in diabetic patients | Darré 2008 [[40](#_ENREF_40)] | Periodontics | Glycated haemoglobin HbA1c | Objective | Periodontal treatment vs. control | 6 |
| Surgical Techniques for the removal of mandibular wisdom teeth | Coulthard 2014 [[41](#_ENREF_41)] | Oral and maxillofacial surgery | Swelling | Subjective | Primary vs. secondary wound closure | 6 |
| Psychological treatment of dental anxiety among adults | Wide Boman 2013 [[42](#_ENREF_42)] | Oral medicine and pathology | Dental anxiety scale | Subjective | Behavioral therapy vs. control | 5 |
| Systemic interventions for recurrent aphthous stomatitis (mouth ulcers) | Brocklehurst 2012 [[43](#_ENREF_43)] | Oral medicine and pathology | Pain | Subjective | Intervention versus control | 5 |
| Psychosocial interventions for the management of chronic orofacial pain | Aggarwal 2011 [[44](#_ENREF_44)] | Oral medicine and pathology | Pain | Subjective | Any psychosocial intervention vs. usual care | 7 |
| Interventions for replacing missing teeth: different types of dental implants | Esposito 2007 [[45](#_ENREF_45)] | Implantology | Bone level | Subjective | Treatment vs. another treatment | 10 |
| Flossing for the management of periodontal diseases and dental caries in adults | Sambunjak 2011 [[46](#_ENREF_46)] | Periodontics | Gingival index | Subjective | Toothbrushing plus flossing vs. toothbrushing alone | 6 |
| Efficacy and co-morbidity of oral appliances in the treatment of obstructive sleep apnea-hypopnea | Hoekema 2004 [[47](#_ENREF_47)] | Orthodontics and dentofacial orthopedics | Apnea-Hypopnea index | Objective | Mandibular repositioning appliance vs. continuous positive airway pressure | 6 |
| Adjunctive photodynamic therapy to non-surgical treatment of chronic periodontitis | Sgolastra 2013 (2) [[48](#_ENREF_48)] | Periodontics | Clinical attachment level | Subjective | Scaling root planing plus antimicrobial photodynamic therapy vs. scaling root planing | 11 |
| Fluoride mouth rinses for preventing dental caries in children and adolescents | Marinho 2003 [[49](#_ENREF_49)] | Dental public health and pediatric dentistry | The decayed, missing, and filled surfaces (DMFS) index | Subjective | Fluoride mouth rinse vs. placebo or no-treatment | 26 |
| The use of enamel matrix derivative alone versus in combination with bone grafts to treat patients with periodontal intrabony defects | Li 2012 [[50](#_ENREF_50)] | Periodontics | Clinical attachment levels | Subjective | Enamel matrix derivative plus bone grafts vs. enamel matrix derivative alone | 5 |
| Treatment of periodontitis improves the atherosclerotic profile | Teeuw 2014 [[51](#_ENREF_51)] | Periodontics | hsCRP levels | Objective | Periodontal treatment vs. control | 13 |
| The effect of cetylpyridinium chloride-containing mouth rinses as adjuncts to toothbrushing on plaque and parameters of gingival inflammation | Haps 2008 [[52](#_ENREF_52)] | Periodontics | Plaque index | Subjective | Cetylpyridinium chloride mouth rinses vs. brushing only | 6 |
| Different powered toothbrushes for plaque control and gingival health | Deacon 2010 [[53](#_ENREF_53)] | Periodontics | Gingival index | Subjective | Side to side vs. rotation oscillation | 6 |
| Treatment of class II molar furcation involvement | Kinaia 2011 [[54](#_ENREF_54)] | Periodontics | Bone level | Subjective | Non-resorbable vs. resorbable membranes | 5 |
| The long-term effect of a mouth rinse containing essential oils on dental plaque and gingivitis | Stoeken 2007 [[55](#_ENREF_55)] | Periodontics | Plaque index | Subjective | Mouth rinse containing essential oils rinse vs. control | 6 |
| Fluoride gels for preventing dental caries in children and adolescents | Marinho 2002 (2) [[56](#_ENREF_56)] | Dental public health and pediatric dentistry | The decayed, missing, and filled surfaces (DMFS) index | Subjective | Fluoride gel vs. placebo or no treatment | 19 |
| Corticosteroids reduce postoperative morbidity after third molar surgery | Markiewicz 2008 [[57](#_ENREF_57)] | Oral and maxillofacial surgery | Late edema | Subjective | Corticosteroids vs. placebo | 6 |
| Pharmacological management of pain during orthodontic treatment | Angelopoulou 2012 [[58](#_ENREF_58)] | Orthodontics and dentofacial orthopedics | Pain | Subjective | Ibuprofen vs. placebo | 6 |
| Interventions for replacing missing teeth: different times for loading dental implants | Esposito 2013 [[59](#_ENREF_59)] | Implantology | Marginal bone level | Subjective | Immediate vs. conventional loading | 8 |
| Fluoride toothpaste efficacy and safety in children younger than 6 years | Wright 2014 [[60](#_ENREF_60)] | Dental public health and pediatric dentistry | The decayed, missing, and filled teeth (DMFT) index | Subjective | Fluoride toothpastes vs. control | 8 |
| The efficacy of bone replacement grafts in the treatment of periodontal osseous defects | Reynolds 2003 [[61](#_ENREF_61)] | Periodontics | Bone level | Subjective | Bone replacement grafts vs. open flap debridement  defects | 18 |
| Secondary versus primary closure techniques for preventing postoperative complications following removal of impacted mandibular third molars | Carrasco-Labra 2012 [[62](#_ENREF_62)] | Oral and maxillofacial surgery | Pain | Subjective | Secondary vs primary closure technique | 7 |
| Evidence that periodontal treatment improves diabetes outcomes | Engebretson 2013 [[63](#_ENREF_63)] | Periodontics | Glycated haemoglobin HbA1c | Objective | Scaling and root planing vs. non-treatment | 5 |
| Primary prevention of dental erosion by calcium and fluoride | Zini 2014 [[64](#_ENREF_64)] | Dental public health | Dental erosion prevention | Objective | Calcium vs. water | 6 |

**References for Appendix 1**

1. Eberhard J, Jepsen S, Jervoe-Storm PM, Needleman I, Worthington HV: Full-mouth disinfection for the treatment of adult chronic periodontitis. Cochrane Database Syst Rev. 2008(1):CD004622.

2. Cairo F, Pagliaro U, Nieri M. Treatment of gingival recession with coronally advanced flap procedures: a systematic review. J Clin Periodontol. 2008, 35(8 Suppl):136-162.

3. Sgolastra F, Petrucci A, Gatto R, Monaco A. Effectiveness of systemic amoxicillin/metronidazole as an adjunctive therapy to full-mouth scaling and root planing in the treatment of aggressive periodontitis: a systematic review and meta-analysis. J Periodontol. 2012, 83(6):731-743.

4. Stoecklin-Wasmer C, Rutjes AW, da Costa BR, Salvi GE, Juni P, Sculean A. Absorbable collagen membranes for periodontal regeneration: a systematic review. J Dent Res. 2013, 92(9):773-781.

5. Sohrabi K, Saraiya V, Laage TA, Harris M, Blieden M, Karimbux N. An evaluation of bioactive glass in the treatment of periodontal defects: a meta-analysis of randomized controlled clinical trials. J Periodontol. 2012, 83(4):453-464.

6. Atieh MA, Ibrahim HM, Atieh AH. Platform switching for marginal bone preservation around dental implants: a systematic review and meta-analysis. J Periodontol. 2010, 81(10):1350-1366.

7. Annibali S, Bignozzi I, Cristalli MP, Graziani F, La Monaca G, Polimeni A. Peri-implant marginal bone level: a systematic review and meta-analysis of studies comparing platform switching versus conventionally restored implants. J Clin Periodontol. 2012, 39(11):1097-1113.

8. Del Fabbro M, Bortolin M, Taschieri S, Weinstein R. Is platelet concentrate advantageous for the surgical treatment of periodontal diseases? A systematic review and meta-analysis. J Periodontol. 2011, 82(8):1100-1111.

9. Davies RM, Ellwood RP, Davies GM. The effectiveness of a toothpaste containing triclosan and polyvinyl-methyl ether maleic acid copolymer in improving plaque control and gingival health: a systematic review. J Clin Periodontol. 2004, 31(12):1029-1033.

10. Kim AJ, Lo AJ, Pullin DA, Thornton-Johnson DS, Karimbux NY. Scaling and root planing treatment for periodontitis to reduce preterm birth and low birth weight: a systematic review and meta-analysis of randomized controlled trials. J Periodontol. 2012, 83(12):1508-1519.

11. Sgolastra F, Petrucci A, Severino M, Gatto R, Monaco A. Lasers for the treatment of dentin hypersensitivity: a meta-analysis. J Dent Res. 2013, 92(6):492-499.

12. Marinho VC, Higgins JP, Sheiham A, Logan S. Combinations of topical fluoride (toothpastes, mouthrinses, gels, varnishes) versus single topical fluoride for preventing dental caries in children and adolescents. Cochrane Database Syst Rev. 2004(1):CD002781.

13. Esposito M, Grusovin MG, Papanikolaou N, Coulthard P, Worthington HV. Enamel matrix derivative (Emdogain(R)) for periodontal tissue regeneration in intrabony defects. Cochrane Database Syst Rev. 2009(4):CD003875.

14. Needleman IG, Worthington HV, Giedrys-Leeper E, Tucker RJ. Guided tissue regeneration for periodontal infra-bony defects. Cochrane Database Syst Rev. 2006(2):CD001724.

15. Berchier CE, Slot DE, Haps S, Van der Weijden GA. The efficacy of dental floss in addition to a toothbrush on plaque and parameters of gingival inflammation: a systematic review. Int J Dent Hyg. 2008, 6(4):265-279.

16. Cunha-Cruz J, Stout JR, Heaton LJ, Wataha JC. Dentin hypersensitivity and oxalates: a systematic review. J Dent Res. 2011, 90(3):304-310.

17. Berchier CE, Slot DE, Van der Weijden GA. The efficacy of 0.12% chlorhexidine mouthrinse compared with 0.2% on plaque accumulation and periodontal parameters: a systematic review. J Clin Periodontol. 2010, 37(9):829-839.

18. Walsh T, Worthington HV, Glenny AM, Appelbe P, Marinho VC, Shi X. Fluoride toothpastes of different concentrations for preventing dental caries in children and adolescents. Cochrane Database Syst Rev. 2010(1):CD007868.

19. Bjordal JM, Bensadoun RJ, Tuner J, Frigo L, Gjerde K, Lopes-Martins RA. A systematic review with meta-analysis of the effect of low-level laser therapy (LLLT) in cancer therapy-induced oral mucositis. Supportive care in cancer 2011, 19(8):1069-1077.

20. Sutherland S, Matthews DC. Emergency management of acute apical periodontitis in the permanent dentition: a systematic review of the literature. J Can Dent Assoc 2003, 69(3):160.

21. Ebrahim S, Montoya L, Busse JW, Carrasco-Labra A, Guyatt GH. The effectiveness of splint therapy in patients with temporomandibular disorders: a systematic review and meta-analysis. J Am Dent Assoc 2012, 143(8):847-857.

22. Marinho VC, Higgins JP, Logan S, Sheiham A. Fluoride varnishes for preventing dental caries in children and adolescents. Cochrane Database Syst Rev. 2002(3):CD002279.

23. Paraskevas S, van der Weijden GA. A review of the effects of stannous fluoride on gingivitis. J Clin Periodontol. 2006, 33(1):1-13.

24. Robinson PG, Deacon SA, Deery C, Heanue M, Walmsley AD, Worthington HV, Glenny AM, Shaw WC. Manual versus powered toothbrushing for oral health. Cochrane Database Syst Rev. 2005(2):CD002281.

25. Marinho VC, Higgins JP, Sheiham A, Logan S. Fluoride toothpastes for preventing dental caries in children and adolescents. Cochrane Database Syst Rev. 2003(1):CD002278.

26. Shi Z, Xie H, Wang P, Zhang Q, Wu Y, Chen E, Ng L, Worthington HV, Needleman I, Furness S. Oral hygiene care for critically ill patients to prevent ventilator-associated pneumonia. Cochrane Database Syst Rev. 2013, 8:CD008367.

27. Lin PY, Cheng YW, Chu CY, Chien KL, Lin CP, Tu YK. In-office treatment for dentin hypersensitivity: a systematic review and network meta-analysis. J Clin Periodontol. 2013, 40(1).53-64.

28. Riley P, Lamont T: Triclosan/copolymer containing toothpastes for oral health. Cochrane Database Syst Rev. 2013, 12:CD010514.

29. Furness S, Worthington HV, Bryan G, Birchenough S, McMillan R. Interventions for the management of dry mouth: topical therapies. Cochrane Database Syst Rev. 2011(12):CD008934.

30. Murphy KG, Gunsolley JC. Guided tissue regeneration for the treatment of periodontal intrabony and furcation defects. A systematic review. Annals of periodontology. 2003, 8(1):266-302.

31. Chambrone L, Chambrone D, Pustiglioni FE, Chambrone LA, Lima LA. Can subepithelial connective tissue grafts be considered the gold standard procedure in the treatment of Miller Class I and II recession-type defects? J Dent 2008, 36(9):659-671.

32. Vignoletti F, Matesanz P, Rodrigo D, Figuero E, Martin C, Sanz M. Surgical protocols for ridge preservation after tooth extraction. A systematic review. Clin Oral Implants Res. 2012, 23 Suppl 5:22-38.

33. Hasson H, Ismail AI, Neiva G. Home-based chemically-induced whitening of teeth in adults. Cochrane Database Syst Rev. 2006(4):CD006202.

34. Lin GH, Chan HL, Bashutski JD, Oh TJ, Wang HL. The effect of flapless surgery on implant survival and marginal bone level: a systematic review and meta-analysis. J Periodontol. 2014, 85(5):e91-103.

35. Emami E, Heydecke G, Rompre PH, de Grandmont P, Feine JS. Impact of implant support for mandibular dentures on satisfaction, oral and general health-related quality of life: a meta-analysis of randomized-controlled trials. Clin Oral Implants Res. 2009, 20(6):533-544.

36. Sahrmann P, Puhan MA, Attin T, Schmidlin PR. Systematic review on the effect of rinsing with povidone-iodine during nonsurgical periodontal therapy. Journal of periodontal research 2010, 45(2):153-164.

37. Poulsen S, Errboe M, Lescay Mevil Y, Glenny AM. Potassium containing toothpastes for dentine hypersensitivity. Cochrane Database Syst Rev. 2006(3):CD001476.

38. Lourenco-Matharu L, Ashley PF, Furness S. Sedation of children undergoing dental treatment. Cochrane Database Syst Rev. 2012, 3:CD003877.

39. Pavia M, Nobile CG, Angelillo IF. Meta-analysis of local tetracycline in treating chronic periodontitis. J Periodontol. 2003, 74(6):916-932.

40. Darre L, Vergnes JN, Gourdy P, Sixou M. Efficacy of periodontal treatment on glycaemic control in diabetic patients: A meta-analysis of interventional studies. Diabetes & metabolism 2008, 34(5).497-506.

41. Coulthard P, Bailey E, Esposito M, Furness S, Renton TF, Worthington HV: Surgical techniques for the removal of mandibular wisdom teeth. Cochrane Database Syst Rev. 2014, 7:CD004345.

42. Wide Boman U, Carlsson V, Westin M, Hakeberg M. Psychological treatment of dental anxiety among adults: a systematic review. Eur J Oral Sci. 2013, 121(3 Pt 2):225-234.

43. Brocklehurst P, Tickle M, Glenny AM, Lewis MA, Pemberton MN, Taylor J, Walsh T, Riley P, Yates JM. Systemic interventions for recurrent aphthous stomatitis (mouth ulcers). Cochrane Database Syst Rev. 2012, 9:CD005411.

44. Aggarwal VR, Lovell K, Peters S, Javidi H, Joughin A, Goldthorpe J. Psychosocial interventions for the management of chronic orofacial pain. Cochrane Database Syst Rev. 2011(11):CD008456.

45. Esposito M, Murray-Curtis L, Grusovin MG, Coulthard P, Worthington HV. Interventions for replacing missing teeth: different types of dental implants. Cochrane Database Syst Rev. 2007(4):CD003815.

46. Sambunjak D, Nickerson JW, Poklepovic T, Johnson TM, Imai P, Tugwell P, Worthington HV. Flossing for the management of periodontal diseases and dental caries in adults. Cochrane Database Syst Rev. 2011(12):CD008829.

47. Hoekema A, Stegenga B, De Bont LG. Efficacy and co-morbidity of oral appliances in the treatment of obstructive sleep apnea-hypopnea: a systematic review. Critical reviews in oral biology and medicine : an official publication of the American Association of Oral Biologists 2004, 15(3).137-155.

48. Sgolastra F, Petrucci A, Severino M, Graziani F, Gatto R, Monaco A: Adjunctive photodynamic therapy to non-surgical treatment of chronic periodontitis. a systematic review and meta-analysis. J Clin Periodontol. 2013, 40(5):514-526.

49. Marinho VC, Higgins JP, Logan S, Sheiham A. Fluoride mouthrinses for preventing dental caries in children and adolescents. Cochrane Database Syst Rev. 2003(3):CD002284.

50. Li W, Xiao L, Hu J. The use of enamel matrix derivative alone versus in combination with bone grafts to treat patients with periodontal intrabony defects: a meta-analysis. J Am Dent Assoc 2012, 143(9).e46-56.

51. Teeuw WJ, Slot DE, Susanto H, Gerdes VE, Abbas F, D'Aiuto F, Kastelein JJ, Loos BG. Treatment of periodontitis improves the atherosclerotic profile: a systematic review and meta-analysis. J Clin Periodontol. 2014, 41(1):70-79.

52. Haps S, Slot DE, Berchier CE, Van der Weijden GA. The effect of cetylpyridinium chloride-containing mouth rinses as adjuncts to toothbrushing on plaque and parameters of gingival inflammation: a systematic review. Int J Dent Hyg. 2008, 6(4).290-303.

53. Deacon SA, Glenny AM, Deery C, Robinson PG, Heanue M, Walmsley AD, Shaw WC: Different powered toothbrushes for plaque control and gingival health. Cochrane Database Syst Rev. 2010(12).CD004971.

54. Kinaia BM, Steiger J, Neely AL, Shah M, Bhola M: Treatment of Class II molar furcation involvement: meta-analyses of reentry results. J Periodontol. 2011, 82(3):413-428.

55. Stoeken JE, Paraskevas S, van der Weijden GA. The long-term effect of a mouthrinse containing essential oils on dental plaque and gingivitis: a systematic review. J Periodontol. 2007, 78(7).1218-1228.

56. Marinho VC, Higgins JP, Logan S, Sheiham A. Fluoride gels for preventing dental caries in children and adolescents. Cochrane Database Syst Rev. 2002(2):CD002280.

57. Markiewicz MR, Brady MF, Ding EL, Dodson TB. Corticosteroids reduce postoperative morbidity after third molar surgery: a systematic review and meta-analysis. J Oral Maxillofac Surg 2008, 66(9).1881-1894.

58. Angelopoulou MV, Vlachou V, Halazonetis DJ. Pharmacological management of pain during orthodontic treatment: a meta-analysis. Orthodontics & craniofacial research 2012, 15(2).71-83.

59. Esposito M, Grusovin MG, Maghaireh H, Worthington HV. Interventions for replacing missing teeth: different times for loading dental implants. Cochrane Database Syst Rev. 2013, 3.CD003878.

60. Wright JT, Hanson N, Ristic H, Whall CW, Estrich CG, Zentz RR. Fluoride toothpaste efficacy and safety in children younger than 6 years: a systematic review. J Am Dent Assoc 2014, 145(2):182-189.

61. Reynolds MA, Aichelmann-Reidy ME, Branch-Mays GL, Gunsolley JC. The efficacy of bone replacement grafts in the treatment of periodontal osseous defects. A systematic review. Annals of periodontology. 2003, 8(1):227-265.

62. Carrasco-Labra A, Brignardello-Petersen R, Yanine N, Araya I, Guyatt G. Secondary versus primary closure techniques for the prevention of postoperative complications following removal of impacted mandibular third molars: a systematic review and meta-analysis of randomized controlled trials. J Oral Maxillofac Surg 2012, 70(8).e441-457.

63. Engebretson S, Kocher T. Evidence that periodontal treatment improves diabetes outcomes: a systematic review and meta-analysis. J Periodontol. 2013, 84(4 Suppl):S153-169.

64. Zini A, Krivoroutski Y, Vered Y. Primary prevention of dental erosion by calcium and fluoride: a systematic review. Int J Dent Hyg. 2014, 12(1).17-24.

65. Higgins JP, Altman DG, Gotzsche PC, Juni P, Moher D, Oxman AD, Savovic J, Schulz KF, Weeks L, Sterne JA. The Cochrane Collaboration's tool for assessing risk of bias in randomised trials. BMJ. 2011, 343.d5928.

66. Furlan AD, Pennick V, Bombardier C, Van Tulder M. 2009 Updated method guidelines for systematic reviews in the cochrane back review group. Spine 2009, 34(18).1929-1941.
