# Supplementary material for: Influence of blinding on treatment effect size estimate in randomized controlled trials of oral health interventions
Source: BMC Med Res Methodol. 2018 May 18;18:42. doi: 10.1186/s12874-018-0491-0 (PMC5960173; doi:10.1186/s12874-018-0491-0)
Supplement: Supplementary file 2 — Appendix 1. This file contains details of the meta-analyses included in the study. (DOCX 131 kb) [file 12874_2018_491_MOESM2_ESM.docx]

| **Appendix 2. Search strategy used in the study** | |  |
| --- | --- | --- |
| **Database** | | **Search Strategy** |
| **PubMed** | | ((systematic review* OR meta-analys*)) AND (dent* OR tooth OR teeth OR orthodon* OR oral surg* OR endodon* OR periodon* OR prosthodon* OR pedodon* OR pediatric* AND dentistry OR paediatric* AND dentistry OR dent* AND public health OR oral pathology) |
| **EMBASE** | | (systematic review* or meta-analys*).mp. [mp=ti, ab, sh, hw, tn, ot, dm, mf, dv, kw, nm, ps, rs, ui] AND (dent* OR tooth OR teeth OR orthodon* OR oral surg* OR endodon* OR periodon* OR prosthodon* OR pedodon* OR pediatric* dentistry OR paediatric* dentistry OR dent* public health OR oral pathology).mp. [mp=ti, ab, sh, hw, tn, ot, dm, mf, dv, kw, nm, ps, rs, ui] |
| **MEDLINE** | | (systematic review* or meta-analys*).mp. [mp=ti, ab, sh, hw, tn, ot, dm, mf, dv, kw, nm, ps, rs, ui] AND (dent* OR tooth OR teeth OR orthodon* OR oral surg* OR endodon* OR periodon* OR prosthodon* OR pedodon* OR pediatric* dentistry OR paediatric* dentistry OR dent* public health OR oral pathology).mp. [mp=ti, ab, sh, hw, tn, ot, dm, mf, dv, kw, nm, ps, rs, ui] |
| **ISI Web of Science** | | Topic=(dent* OR tooth OR teeth OR orthodon* OR oral surg* OR endodon* OR periodon* OR prosthodon* OR pedodon* OR pediatric* AND dentistry OR paediatric* AND dentistry OR dent* AND public health OR oral pathology) AND Topic=(systematic review* OR meta-analys*) |
| **Cochrane Database of Systematic Reviews** | | (systematic review* or meta-analys*).mp. [mp=ti, ab, sh, hw, tn, ot, dm, mf, dv, kw, nm, ps, rs, ui] AND (dent* OR tooth OR teeth OR orthodon* OR oral surg* OR endodon* OR periodon* OR prosthodon* OR pedodon* OR pediatric* dentistry OR paediatric* dentistry OR dent* public health OR oral pathology).mp. [mp=ti, ab, sh, hw, tn, ot, dm, mf, dv, kw, nm, ps, rs, ui] |
| **HealthSTAR** | | (systematic review* or meta-analys*).mp. [mp=ti, ab, sh, hw, tn, ot, dm, mf, dv, kw, nm, ps, rs, ui] AND (dent* OR tooth OR teeth OR orthodon* OR oral surg* OR endodon* OR periodon* OR prosthodon* OR pedodon* OR pediatric* dentistry OR paediatric* dentistry OR dent* public health OR oral pathology).mp. [mp=ti, ab, sh, hw, tn, ot, dm, mf, dv, kw, nm, ps, rs, ui] |
